# World Antimalarial Resistance Network (WARN) II: In vitro antimalarial drug susceptibility

**DOI:** 10.1186/1475-2875-6-120

**Published:** 2007-09-06

**Authors:** David J Bacon, Ronan Jambou, Thierry Fandeur, Jacques Le Bras, Chansuda Wongsrichanalai, Mark M Fukuda, Pascal Ringwald, Carol Hopkins Sibley, Dennis E Kyle

**Affiliations:** 1 Parasitology Program, Naval Medical Research Center Detachment, Lima, Peru; 2 Laboratoire d'immunologie Clinique et Parasitaire, Institut Pasteur, Dakar; 3 Unité d'Immunologie Moléculaire des Parasites, Institut Pasteur, Paris, France; 4 Hospital Bichat Claude Bernard, Paris, France; 5 U.S. Naval Medical Research Unit No. 2 (NAMRU-2), Jakarta, Republic of Indonesia; 6 Department of Immunology and Medicine, United States Army Medical Component, Armed Forces Research Institute of Medical Sciences, Bangkok, Thailand; 7 Global Malaria Programme, World Health Organization, 20 Av. Appia, 1211 Geneva 27, Switzerland; 8 Department of Genome Sciences, University of Washington, Seattle, WA, USA; 9 Department of Global Health, College of Public Health, University of South Florida, Tampa, FL, USA

## Abstract

Intrinsic resistance of *Plasmodium falciparum *is clearly a major determinant of the clinical failure of antimalarial drugs. However, complex interactions between the host, the parasite and the drug obscure the ability to define parasite drug resistance in vivo. The in vitro antimalarial drug susceptibility assay determines ex-vivo growth of parasite in the presence of serial drug concentrations and, thus, eliminates host effects, such as drug metabolism and immunity. Although the sensitivity of the parasite to various antimalarials provided by such a test provides an important indicator of intrinsic parasite susceptibility, there are fundamental methodological issues that undermine comparison of in vitro susceptibility both between laboratories and within a single laboratory over time. A network of laboratories is proposed that will agree on the basic parameters of the in vitro test and associated measures of quality control. The aim of the network would be to establish baseline values of sensitivity to commonly used antimalarial agents from key regions of the world, and create a global database, linked to clinical, molecular and pharmacology databases, to support active surveillance to monitor temporal trends in parasite susceptibility. Such a network would facilitate the rapid detection of strains with novel antimalarial resistance profiles and investigate suitable alternative treatments with retained efficacy.

## Background

In the last five years, new antimalarial drug combinations have been deployed rapidly in a wide range of settings. Most of these combinations contain an artemisinin derivative. Although the artemisinin derivatives retain excellent efficacy, the selection of resistance to the artemisinins is only a matter of time. Since treatment courses are generally short, the artemisinin component is dependent upon the partner drug for adequate clinical efficacy. When resistance to the latter reaches a critical level, the efficacy of the ACT falls, and it is no longer a suitable treatment for malaria. Thus, it is crucial to establish systems for detection of the earliest possible signs of resistance to both components of ACTs. Because validated molecular markers are lacking for resistance to artemisinins and because effective partner drugs used in ACTs will mask early clinical indicators of resistance, in vitro surveillance will be the critical surveillance tool for the emergence or artemisinin resistance.

Longitudinal *in vitro *analysis of the susceptibility of *Plasmodium falciparum *strains to antimalarial drugs has three important attributes. First, this approach allows the response of clinical isolates to individual drugs to be assayed, unmodified by important host factors that influence drug efficacy in vivo. This capacity is crucial because it allows surveillance for resistance to both components of an ACT. Second, the progressive decline in drug sensitivity of isolates from the same site is likely to be the most sensitive method to identify incipient resistance in the parasite population [1]. Finally, strains with reduced antimalarial susceptibilities can then be established in continuous culture to provide the tools needed to investigate novel molecular mechanisms of resistance and for tests of susceptibility to other antimalarial agents.

Despite its obvious value, the wide variance associated with the estimates derived from the in vitro assay makes the comparison of data between laboratories problematic. The consortium that met at the Wellcome Trust Sanger Centre in October 2006 proposed that a network of regional laboratories should be established with a common test protocol and quality control methodology. Such a network would link with other established surveillance systems, but provide an additional global perspective within a novel initiative: the World Antimalarial Resistance Network (WARN). The in vitro module of the WARN database will identify the key parameters that can be standardized sufficiently to overcome the major factors responsible for inter- and intra-laboratory variability. This would minimize the bias between geographically separate research sites and identify temporal trends that could reveal the emergence of strains of plasmodia resistant to the drugs that are now being used widely.

## Rationale

The goal of the WARN database is to establish a worldwide system for collating information on resistance to antimalarial drugs. Testing for antimalarial in vitro sensitivity phenotypes from diverse endemic regions to drugs in use or in development is a crucial component of this network, for the following reasons: (1) the response of a patient to drug treatment is complex, and (2) reflects host factors as well as the intrinsic response of the parasites to the drug. Analysis of the parasite response to the drug in vitro minimizes host factors, and provides a measure of fundamental parasite antimalarial sensitivity.

1. The in vitro analysis produces a quantitative estimate of the effectiveness of the drug on the growth of the parasite. Longitudinal studies and comparison of the in vitro parasite responses, within a defined human population can reveal trends in sensitivity to a particular drug and, thus, the earliest possible warning of resistance that is developing in local parasite populations.

2. The introduction of combination therapy for malaria is a significant advance in antimalarial policy, but when therapeutic failures are observed, there is no straightforward way to determine which drug may be responsible. In vitro analysis allows each component of a combination to be tested independently.

3. Many different ACTs are currently being introduced or developed, but some of the long-lived partner drugs are chemically similar to each other and to drugs in current use. In vitro analysis of cross-resistance among potential new drugs is crucial to avoid development or introduction of drugs to which parasites are already partially or significantly resistant.

4. The reemergence of sensitive phenotypes necessitates the continuation to monitor older therapies that may have high drug resistance in a given location. As seen with the studies with chloroquine in Malawi [2], by removing the drug pressure for an extended period of time, older but highly effective therapies can once again be used as first line treatment.

5. Establishment of stable reference lines with known drug sensitivity profiles in vitro is a key component of the proposed network.

a. These lines allow verification of phenotypes in more than one laboratory.

b. Preservation can establish an archive of parasites that define the baseline for sensitivity to a newly introduced drug.

Despite considerable efforts, molecular markers of resistance to most components of the ACTs have not yet been identified. Parasites with verified resistant phenotypes can provide the samples needed for identification of the genetic determinants of drug resistance to particular drugs.

## Challenges

### Standardization of procedures

The original WHO in vitro micro-technique was developed by Karl Rieckmann in 1978 to define the minimum inhibitory concentration (MIC) of malaria parasites from clinical isolates [3]. In 1979, Desjardins and colleagues [4] described a modification that measured tritiated hypoxanthine incorporation and the emphasis of the final estimate of susceptibility shifted to the IC_50_. The latter, defined as the concentration of a drug required to inhibit parasite growth by 50% compared with the same sample grown without drug, is now the standard measure of antimalarial in vitro susceptibility. Subsequent modifications have proposed alternative methods of quantifying parasite growth such as ELISA based (e.g. pLDH or HRPII), or DNA detection (SYBG green I or Pico green) detection. Numerous variations in the details of these protocols currently make it impossible to compare these IC_50 _values directly from one laboratory to another [5,6]. At the workshop in October 2006, a solution to overcome these problems was suggested that involved the adoption of two important principles.

First, that the culture system that underlies the in vitro assay be standardized with agreement on: (1) the culture medium, (2) the initial haematocrit, (3) the initial % parasitaemia, and (4) the duration of parasite-drug contact. The second major principle is the adoption of standardized quality controls.

## Methods

A common set of reference strains whose genotype and phenotype are known would be used in all laboratories. The IC_50 _values for drugs to be assayed would be determined routinely on these strains and reported in parallel with the values determined for the experimental isolates. Reference strains with known sensitivity to many drugs are available from the MR4 collection [7]and other strains could be added as they are defined, or as they become relevant to studies in a particular region. The WHO has actively supported standardization of in vitro tests and has provided test kits for use in the field [8,9]. To assure drug quality and appropriate dosing, 96 well microtitre plates with known concentrations of mefloquine, quinine, chloroquine, dihydroartemisin, artemisinin and monodesethylamodiaquine, (the active metabolite of amodiaquine) have been available. However, many laboratories produce their own plates for assay of these drugs, and some components of ACTs have not yet become generally available. The proposed network would assure that relevant drugs and metabolites, together with specific advice on the preparation of test plates, were provided to the regional laboratories. Many of these newer drugs have limited solubility and lose potency rapidly so prepared plates do not have a long shelf life. Under these circumstances, the inclusion of reference strains in the overall in vitro assessment will ensure that the drugs used are of appropriate quality and activity, and that the test plates are uniformly prepared according to set guidelines. Inclusion of regional or global controls will help to eliminate laboratory bias, ensure more consistent results, and enable comparison of data between laboratories worldwide.

## Structure

### Local and regional networks

The establishment of a network of cooperating laboratories and regional reference laboratories will meet these requirements most effectively. The centres would conduct periodic testing on the in vitro plates using control isolates. Centres could also prepare, store and distribute the reference isolates to ensure the integrity of the strains and the stability of their phenotypes. There will certainly be inter-laboratory variation since small differences in culture conditions will remain, even with a standard protocol, but this variation would be minimized and controlled by comparison of tests on the same reference strains. If a laboratory reported a reference strain test with a value lying outside the accepted range of the IC50, this would trigger a response from the regional centre to address the problem.

#### Methods for measuring growth inhibition

In contrast to the agreement that common protocols are necessary for in vitro culture and in vitro test conditions, the group proposed that many different methods for measuring the growth of the parasites were feasible, and would still produce comparable data. A widely used method, incorporation of ^3^H-hypoxanthine into high molecular weight nucleic acids, has been the "gold standard" for some time, but ELISA-based methods, fluorescent dyes that intercalate into DNA and real time PCR are gaining advocates, as well [10-15]. Each method currently described in the literature has supporters and detractors and new ones will surely be developed. Some methods are more adapted to regional centres, and others may be more appropriate in smaller laboratories. The group proposed that centres could choose the output method most appropriate to their equipment and personnel, and the adoption of reference strains can assure comparability of the results obtained with the different output assays. The most important point about the assay method is consistency. When in vitro culture conditions are maintained, comparable test data are produced, even if different methods are used to quantify parasite growth. However, once a method is chosen it is crucial that subsequent longitudinal studies conducted in the same area be done with the same test system, and any change to a different method be validated with the previous approach. This consistent approach will permit detection of small increases in the IC_50 _values of local isolates, and can serve as an early indication of resistance to the drug in question.

### Choice of isolates to be assayed

Early detection of resistance to drugs currently in use will require that baseline parasite chemosensitivity of current isolates from endemic regions is established, both in absolute terms as well as the distribution and range of values expected from a particular laboratory assay. The testing of parasites freshly isolated from patients will provide key data. However, it is also critical to establish contemporary lines in continuous culture, and to freeze aliquots of those lines for future reference. This will allow putatively resistant isolates to be studied at local and regional level facilities. Careful verification (or refutation) of resistance in local isolates is, perhaps, the most important role for in vitro studies [16]. Patients fail treatment for a variety of reasons, even when the infecting parasites are still sensitive to the drug: poor drug absorption, atypical metabolism, the presence of co-infections and many other factors. These parameters are especially important but often poorly understood for the newer ACTs, [17-20], and it is crucial to determine whether parasite resistance to the treatment is the actual cause of clinical failure. The determination of the in vitro sensitivity of the putatively resistant parasites is the most direct approach.

In vitro data from retrospective studies of previously cultured parasites can also be an important and useful part of the proposed database. Archiving of current isolates with dependable data on their sensitivity to a wide range of drugs will also allow each region to establish baseline values and thus to follow more effectively the trends in sensitivity to the drugs in use [1,18,21-23]. Documentation of rising IC_50 _values over the original background is a clear warning of developing resistance. In aggregate, data from these disparate isolates can provide baseline platforms for comparison of temporal trends as surveillance is developed and extended.

### Data handling, quality and reporting

**Figure 1:**
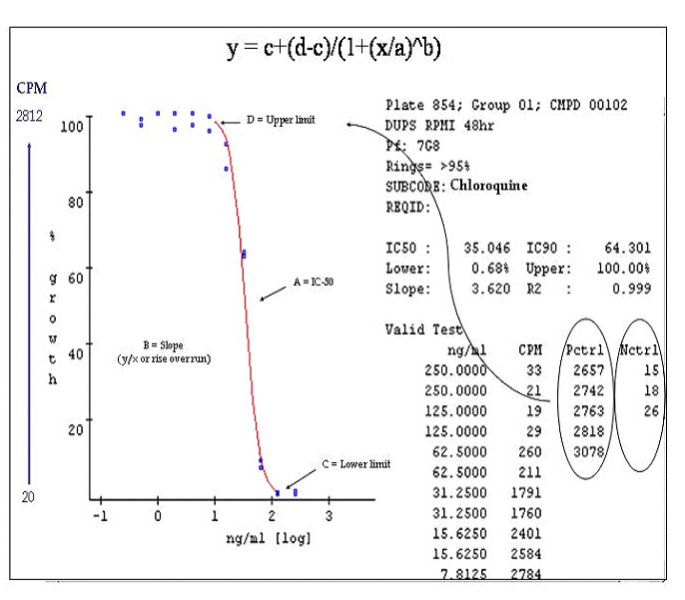
**Output of the raw data from an in vitro experiment**. This is an example of output from tools that will be freely available on the in vitro module of the web site.

The overall database will be created using individual patient records. The in vitro test for one parasite strain/one drug constitutes a series of measurements of parasite growth, over the drug/parasite contact period, in a range of drug concentrations. Normally, the 8 wells of a 96-well micro-culture plate are sufficient to generate a dose-response curve from which the essential output, the IC_50_, can be determined. Whatever the method chosen to measure parasite growth, collection of data into a local electronic data base will allow the efficient archiving and analysis of the data for each drug-parasite test. A number of analysis programmes are available [24] and the in vitro module of this database will have such a programme on the web site. Figure 1 shows the output from one such programme, which includes a display of the input data, the resulting dose-response curve and the formula for calculating the IC_50 _value. Key parameters of the graph are calculated, so that the quality of the data can be readily assessed. The IC_50 _(or any other proportional degree of inhibition, like the IC_90_) is also calculated using the logistic dose response, using a four-parameter equation including the IC_50 _– Slope, the E_min _(lower limit or asymptote) and the E_max _(upper limit or asymptote).

In addition, calculation of the geometric mean of the IC_50 _values from a group of isolates and the standard deviation or 95% confidence interval will be readily managed using the programme on the web. This will facilitate the analysis of data by individual groups, but the programme will not automatically enter the data into the central database. That action will require specific authorization from the group that has generated the data and from the database manager. However, this system should allow a straightforward path for both data and the analysis to be submitted for entry into the central database. Inclusion of this kind of information, along with the data and calculations of the same parameters for the reference strains, determined in parallel, should permit data from different sites and different times to be compared.

The database would not set a cut off for sensitivity vs. resistance of the isolates assayed for particular drugs. These designations have created confusion [25], and it is clear that even when data are collected in a consistent way, the IC_50 _values for a set of strains usually represent a continuous distribution rather than the bimodal distribution suggested by the classification. However, by linking data with in vivo results, pharmacokinetics, and when possible, molecular markers of resistance, researchers can start to set limits of sensitivity. However, arbitrary cut offs applied to in vitro data are probably not useful.

Tools included in the in vitro module of the database will have software programmes that make analyses of subsets of the data possible by any user. The database will be designed so that all entries can be queried with common algorithims. For example, the inclusion of individual data would make it possible to query the database to determine the rates of change in IC_50 _values for a particular drug in isolates from different regions. Currently, most of the information on the speed with which resistance evolves to particular drugs derives from Southeast Asian sites [13,26-28]. It is not at all clear how those rates might predict the spread of resistance in sites with very different characteristics and levels of transmission [29]. The proposed network will collect the necessary data and the database will collate the information to allow questions of this kind to be productively addressed.

The database is designed for open access on the web, and it will be most valuable if data are submitted as rapidly as they are generated. Access to the tools for data entry and analysis will be freely available on the web site, and this will provide an incentive for groups to submit data to the database manager for inclusion in the database. This approach will facilitate the preparation for publication of the data that have been collected by a group, and assure that the important professional work of in vitro surveillance is rewarded. Although the database will include information from patient isolates, the entries will be anonymous, and the linkage to any patient information will be designed to assure patient anonymity.

## Benefits

### Rapid identification of putative resistant isolates

In vitro assay of patient isolates and comparison with the sensitivity of reference strains to the same drugs is still the most direct way to determine whether parasite resistance is responsible for an increase in drug treatment failures. If a decline in clinical efficacy of an ACT is observed, in vitro testing of patient isolates will be required to determine whether host factors or an intrinsic decline in parasite susceptibility underlie the observation [30,31]. The proposed network must be organized and activated now, so that baseline sensitivity to the various ACT partner drugs can be clearly defined. In vitro testing is the only system that is presently available to provide clear early warning of impending resistance to the components of ACTs. A network of laboratories that can produce high quality in vitro data from many different endemic areas and make these data accessible in an open database can provide the surveillance that is required.

### Definition of molecular markers for resistance to ACT components

In vitro assays of drug sensitivity to chloroquine and sulphadoxine-pyrimethamine and mefloquine have played a key role in defining the molecular markers responsible for resistance to these drugs. The genetic changes associated with resistance were identified by comparison of the genotypes of parasites with sensitive and resistant phenotypes, but the loci responsible for resistance were identified long after clinical resistance to the drugs had risen to unacceptably high levels (reviewed in [13,32-34]). Definitive molecular markers for drug resistance to the different artemisinin derivatives being used, or indeed to any of the partner drugs, except mefloquine, are not available [19]. Because of the very short half-life of artemisinins, they have been paired in combinations with longer-lived partners for longer drug action and prophylaxis against reinfecting parasites. This approach means that parasites are then exposed in vivo to these partner drugs alone for an extended period, and selection of resistance to the partner drugs is far more likely than to the artemisinin. There are already hints that this is occurring [19,35-37]. Rapid identification of resistance to a long-lived partner will allow a new partner to be chosen, protecting the artemisinin component. This assumes, of course, that there is an active "pipeline" of potential partners available when the need arises.

In vitro identification of a series of parasite lines from natural isolates stably resistant to artemisinins and to their partner drugs, lumefantrine, mefloquine, pyronaridine, piperaquine and amodiaquine is the first step to define the genetic basis of resistance to each class of drug. Comparison of the genomes of these resistant parasites with related lines that remain sensitive to the drug will allow genetic differences to be identified and loci responsible for the resistance to be validated. A reference genome of the 3D7 *P. falciparum *is available [38], and single nucleotide polymorphisms [39-41], small insertions and deletions [42] and changes in copy number [43] in patient isolates and strains established from them can now be compared relatively rapidly with reference genomes. This whole genome approach readily identified the locus that is principally responsible for resistance to chloroquine [44] and this is the most direct approach to identify loci with associations to resistance to the various components of ACTs. Molecular markers associated with resistance to any of the components of ACTs could provide a second key early warning of the emergence and spread of resistance to these valuable drugs.

### Linkage to other modules of the WARN database

The World Antimalarial Resistance Network aims to create an individual level database containing information on clinical, in vitro, pharmacokinetic and molecular markers of antimalarial resistance. The system is described in more detail in the paper on the clinical module of the database and on the web site where the prototype database resides [45]. There will be some instances where both the clinical outcome of treatment and contemporary determination of in vitro sensitivity to drugs will be available. Moreover, the pharmacokinetic response of a patient to the treatment drug may be linked from that module (a sub-set of the main database), as well. When complementary information like this is available on an isolate, the records will be linked so that pharmacokinetic and pharmacodynamic information can be correlated. However, the bulk of the data on in vitro analysis is unlikely to be linked to a particular patient record. In those cases, individual records will still be used, and information on the time and place of collection of the isolate will be included. Molecular markers of resistance to particular drugs will also be determined for many of the individual isolates that are tested for chemosensitivity in vitro, and will be linked, through the database structure, to the in vitro test results.

### Understanding geographic and temporal trends in parasite resistance

Currently, in vitro data on antimalarial drug resistance are available only for a few locations. The absence of regional information and the lack of coordinated data over a long time period make it almost impossible to follow trends that could give early warning that resistance to the antimalarial drugs in use is emerging. This network and the associated database will be a common source of data on the in vitro response to drugs of parasites from many endemic areas. The database will provide programmes on the web site that will facilitate the analysis and presentation of trends in in vitro sensitivity from all malarial regions and on a real-time basis. Those who advise policy makers will then have the tools needed to present a coherent picture of drug resistance in their country and region. This feature should make it possible to provide evidence to those responsible for changing drug policy when increasing resistance is observed.

## Conclusion

The introduction of ACTs has made a dramatic impact on malaria treatment in many countries [46], and there is progress in solving the various economic and logistical problems that have slowed their introduction in some regions [47]. Currently, these drugs are effective almost everywhere, but there are already hints that resistance to artemisinins is emerging [30] and resistance has already compromised the efficacy of some ACT partner drugs. It is crucial to quickly devise and activate a comprehensive plan for monitoring closely ACT drug effectiveness. WARN sets out to do just that. The clinical components of the database will provide much needed data on drug efficacy, but the malaria community cannot afford to wait until clinical failure of these drugs arises. The experience with chloroquine and sulphadoxine-pyrimethamine is clear: if action is taken only after clinical failures are common, it will be too late to save these valuable drugs. The in vitro network will provide the most sensitive bellwether of the emergence of resistant parasites: rising IC_50 _values of contemporary parasites. Moreover, parasites with drug resistant phenotypes in vitro are the key to using modern genomic comparisons to identify the genetic changes that underlie the resistance. There is no time to waste.

## Authors' contributions

DJB wrote the first draft of the manuscript, DEK chaired the in vitro subgroup, and RJ, TF, JLB, CW, MMF and PR contributed to the discussions and edited the manuscript.
